# Negative thermal expansion and broad band photoluminescence in a novel material of ZrScMo_2_VO_12_

**DOI:** 10.1038/srep24832

**Published:** 2016-04-21

**Authors:** Xianghong Ge, Yanchao Mao, Xiansheng Liu, Yongguang Cheng, Baohe Yuan, Mingju Chao, Erjun Liang

**Affiliations:** 1 College of Physical Science and Engineering & Key Laboratory of Materials Physics of Ministry of Education of China, Zhengzhou University, Zhengzhou 450052, China; 2 Zhongyuan University of Technology, College of Science, Zhengzhou 450007, China; 3 Key Laboratory of Photovoltaic Materials of Henan Province and School of Physics & Electronic, Henan University, Kaifeng 475004, China

## Abstract

In this paper, we present a novel material with the formula of ZrScMo_2_VO_12_ for the first time. It was demonstrated that this material exhibits not only excellent negative thermal expansion (NTE) property over a wide temperature range (at least from 150 to 823 K), but also very intense photoluminescence covering the entire visible region. Structure analysis shows that ZrScMo_2_VO_12_ has an orthorhombic structure with the space group *Pbcn* (No. 60) at room temperature. A phase transition from monoclinic to orthorhombic structure between 70 and 90 K is also revealed. The intense white light emission is tentatively attributed to the n- and p-type like co-doping effect which creates not only the donor- and acceptor-like states in the band gap, but also donor-acceptor pairs and even bound exciton complexes. The excellent NTE property integrated with the intense white-light emission implies a potential application of this material in light emitting diode and other photoelectric devices.

Thermal expansion is a popular phenomenon while thermal contraction or negative thermal expansion (NTE) is rarely seen in nature. It is well known that most materials expand on heating and contract on cooling with very different rates, which has caused a lot of troubles in modern technologies, such as fatigues, delamination, cracking and temperal or permanent inactivation of devices. To overcome thermal expansion or the mismatch in the coefficients of thermal expansion (CTE) in different materials has long been a difficult problem. The discovery of the NTE in α-ZrW_2_O_8_ within a large temperature range threw a light on solving the problem and stimulated much interests in the NTE phenomenon^1^,^2^,^3^,^4^,^5^,^6^,^7^. Since then, plenty of NTE materials has been discovered, such as open framework structure oxides A_2_M_3_O_12_ (A = transition metal, M = W, Mo)^8^,^9^,^10^,^11^,^12^,^13^,^14^, AMgM_3_O_12_ (A = Zr, Hf. M = W, Mo)^15^,^16^,^17^,^18^,^19^,^20^ and ZrV_2_O_7_^21^,^22^, cyanides M(CN)_2_ (M = Zn, Cd) and Ag_3_[Co(CN)_6_]^23^,^24^, fluorides ScF_3_ and ZnF_2_^25^,^26^, anti-perovskite manganese nitrides Mn_3_AN (A = Zn, Ga and Ge, etc.)^27^,^28^,^29^,^30^,^31^,^32^, perovskite structure PbTiO_3_^33^, BiNiO_3_^34^ and LaCu_3_Fe_4_O_12_^35^, and high quartz structure beta-Li_2_Al_2_SiO_6_^36^, beta-LiAlSiO_4_^37^, and keatite^38^, etc. The NTE mechanisms in the open framework structure arise mainly from phonon anharmonicity^3^,^6^,^11^,^21^,^23^,^24^,^25^ while in others most related to phase transition, such as magnetovolume effect in anti-perovskite manganese nitrides, ferroelectric-to-paraelectric phase transition in PbTiO_3_ and temperature-induced intersite charge transfer in LaCu_3_Fe_4_O_12_ and BiNiO_3_.

Although a number of NTE materials have been reported, they are still very limited and far from meeting the requirements of various devices due to the property limitations of the known NTE materials, such as narrow temperature scope or unsuitable temperature range of the NTE, hygroscopicity and improper phase-transition temperature in some materials, etc. In fact, applicable NTE materials in engineering are much fewer up to date though many efforts have been made on adjusting the properties of the NTE materials. Developing novel materials with excellent NTE property and additional functionality is of particular importance both scientifically and technically.

In this paper, we report a novel material with the formula of ZrScMo_2_VO_12_, which possesses excellent continuous NTE property in a wide temperature range and exhibits intense wide-band photoluminescence (PL) covering the entire visible light region. Structure analysis shows that ZrScMo_2_VO_12_ crystallizes in an orthorhombic structure with group of *Pbcn* (60). Temperature-dependent photoluminescence and Raman spectroscopic studies indicate a phase transition between 70~98 K. A close relationship between the thermal expansion property and PL is also observed. To the best of our knowledge, the material with a formula ZrScMo_2_VO_12_ has not been reported. Its excellent NTE property integrated with the intense white-light emission functionality suggests a potential application of this material in light emitting diode and other photoelectric devices.

## Results

**Figure 1:**
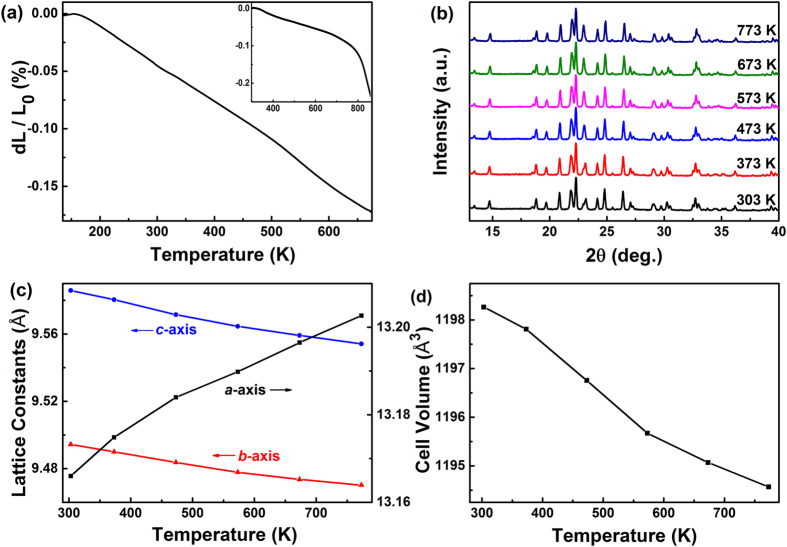
Relative length change and temperature dependent XRD. (**a**) Relative length changes of the ZrScMo_2_VO_12_ cylinder with increasing temperature from 135 to 673 K and from RT to 873 K (Inset); (**b**) Temperature dependent XRD patterns of ZrScMo_2_VO_12_; (**c**) Change in lattice constants and (**d**) cell volume of ZrScMo_2_VO_12_ with temperature.

The NTE property of ZrScMo_2_VO_12_ is investigated with dilatometer. Figure 1a and the inset show the relative length changes of a sintered ZrScMo_2_VO_12_ cylinder with increasing temperature in the low and high temperature regions, respectively. It can be clearly seen that the sintered ZrScMo_2_VO_12_ cylinder exhibits a continuous shrinkage from about 135 to 873 K. This result demonstrates an excellent NTE property of the material with a wide temperature range covering room temperature (RT). The CTEs are calculated to be −3.25 × 10^−6^ K^−1^ (150–675 K) and −2.20 × 10^−6^ K^−1^ (293–823 K), respectively.

The linear CTE measured by a dilatometer reveals the average property of thermal expansion or contraction of a bulk material. In order to investigate the axial thermal expansion or contraction, namely the intrinsic change of the crystal lattice with temperature, we carried out temperature-dependent X-ray diffraction (XRD) measurements as shown in Fig. 1b. The lattice constants and volume at each temperature were calculated from the corresponding XRD pattern by the same method. It is shown that the *b-* and *c*-axes contract while the *a*-axis expands continuously with increasing temperature (Fig. 1c), leading to a thermal shrinkage in volume (Fig. 1d). The CTEs of the *a-*, *b-*, and *c-*axes are calculated to be *α*_*a*_ = 5.93 × 10^−6^ K^−1^, *α*_*b*_ = −5.43 × 10^−6^ K^−1^ and *α*_*c*_ = −7.05 × 10^−6^ K^−1^, respectively. This gives rise to a volume CTE *α*_*v*_ = −6.57 × 10^−6^ K^−1^ and a linear CTE α_l_ = −2.19 × 10^−6^ K^−1^ from RT to 773 K, which agrees well with the results measured by dilatometry.

**Figure 2:**
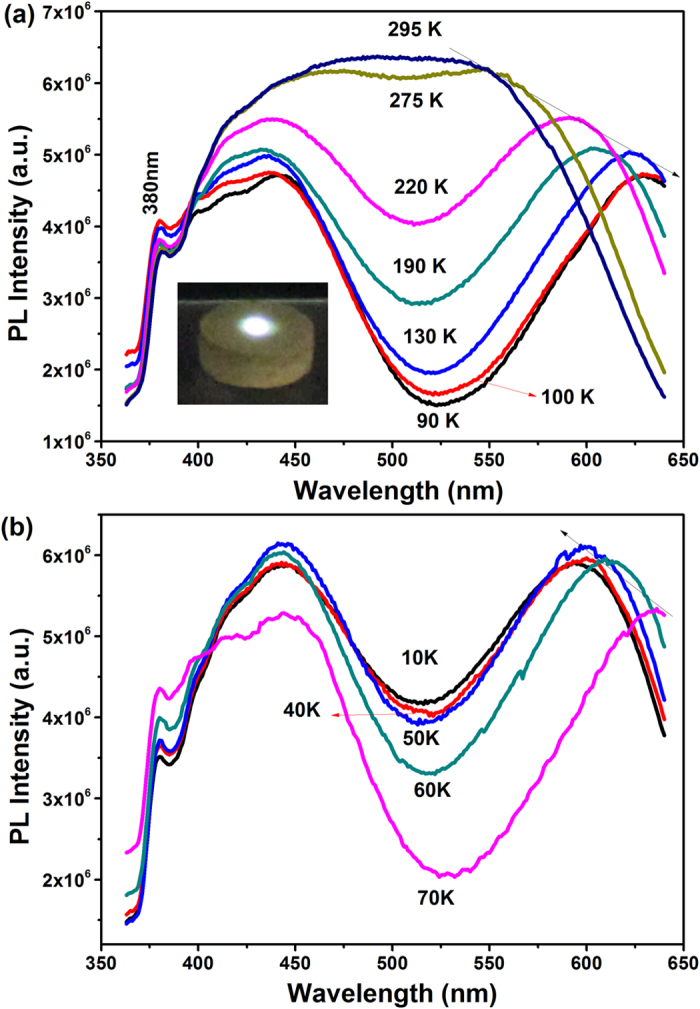
Temperature dependent PL. (**a**,**b**) Temperature-dependent PL spectra of ZrScMo_2_VO_12_ with 345 nm excitation. The optical photograph in the inset of (**a**) was taken under irradiation with 345 nm.

The optical property of ZrScMo_2_VO_12_ is also investigated by temperature-dependent PL spectra with excitation of 345 nm as shown in Fig. 2a,b. The inset of Fig. 2a is the optical photograph of the ZrScMo_2_VO_12_ sample under an excitation with wavelength of 345 nm. The ZrScMo_2_VO_12_ sample exhibits very intense white light emission which can be clearly seen by naked eyes.

## Discussion

**Figure 3:**
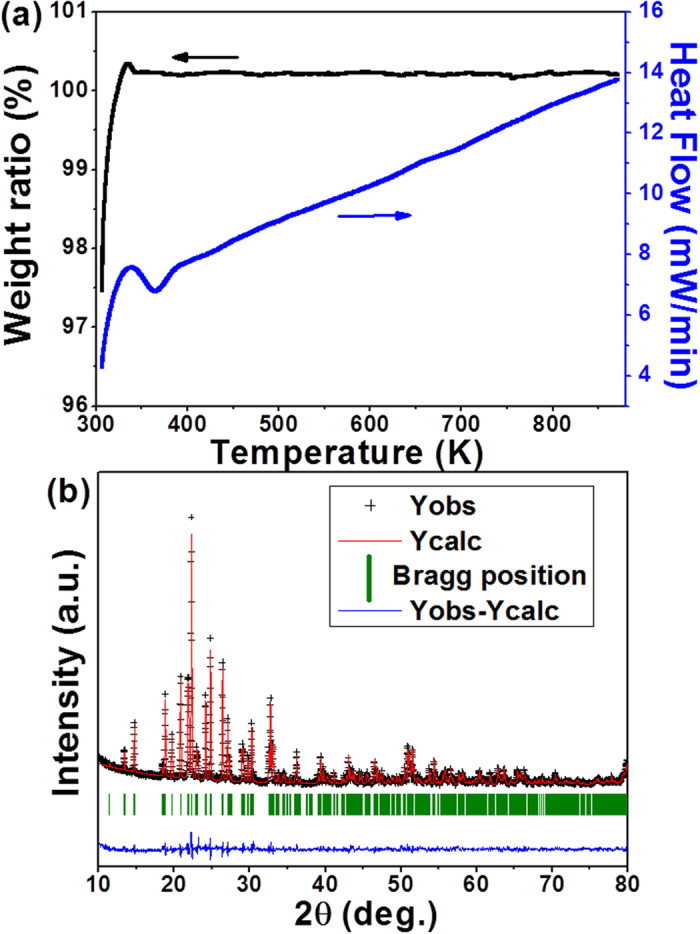
TG/DSC curves and XRD pattern refinement. (**a**) TG/DSC curves of ZrScMo_2_VO_12_ from RT to 873 K; (**b**) XRD pattern of the sample at RT and the result of the Rietveld analysis (R_p_ = 8.08%, R_wp_ = 11.92%, and R_exp_ = 5.09%). The “+” signs represent the observed profile. The solid line represents the calculated profile. Vertical bars indicate the position of Bragg peaks for this phase. The lowest curve is the difference between the observed and calculated patterns.

It was demonstrated that the relative length change of a material is very sensitive to crystal water releasing during temperature increasing^9^,^20^. From the curves of linear thermal expansion, we can figure out that ZrScMo_2_VO_12_ is an excellent NTE material without hygroscopicity and phase transition in the investigated temperature range because either hygroscopicity or phase transition would result in abnormal changes of the linear thermal expansion curves during temperature increasing^9^,^20^. In order to confirm this, we performed the differential scanning calorimetry (DSC) and thermogravimetry (TG) measurements. Figure 3a shows the DSC and TG plots of ZrScMo_2_VO_12_ from RT-873 K. The dip near 375 K is an instrumental artifact. Neither obvious endothermic/exothermic peaks nor evident weight loss appear in the corresponding curves, confirming that ZrScMo_2_VO_12_ exhibits neither phase transition and nor hygroscopicity from 400–873 K.

The crystal structure is refined by Rietveld analysis. Figure 3b shows the result of the Rietveld analysis for the XRD pattern. The analysis shows that ZrScMo_2_VO_12_ adopts an orthorhombic structure with space group of *Pbcn* (No. 60) at RT. The cell lattice parameters are calculated to be *a* = 13.1660, *b* = 9.4944 and *c* = 9.5859, respectively, with the acceptable values of R_p_ = 8.08%, R_wp_ = 11.92% and R_exp_ = 5.09%.

**Figure 4:**
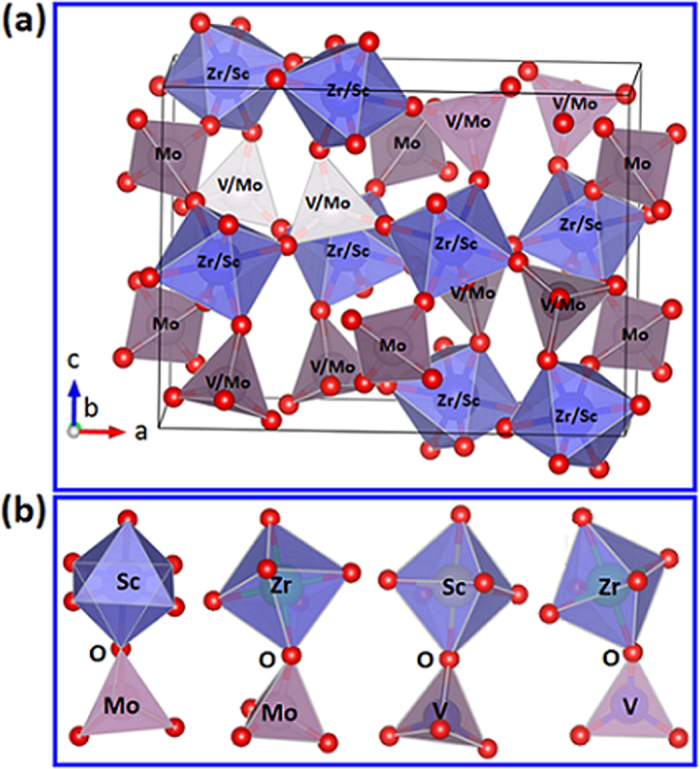
Schematic diagrams of ZrScMo_2_VO_12_ and Zr/Sc-O-Mo/V polyhedral structures. (**a**) Schematic diagram of ZrScMo_2_VO_12_ building block (red sphere indicates oxygen atom); (**b**) Schematic diagrams of Zr/Sc-O-Mo/V polyhedral structures in ZrScMo_2_VO_12_ (red balls indicate oxygen atoms).

Detailed mechanism of the NTE in ZrScMo_2_VO_12_ requires more precise analyses and first principles calculations. Here we give only a rough understanding. Figure 4 presents the schematic diagram of ZrScMo_2_VO_12_ building block depending on the XRD refinement result. In this structure, Zr^4+^ and Sc^3+^ are octahedrally while Mo^6+^ and V^5+^ are tetrahedrally coordinated with oxygen, forming corner-sharing ZrO_6_/ScO_6_ octahedra and MoO_4_/VO_4_ tetrahedra as in A_2_M_3_O_12_ family. Therefore the NTE in ZrScMo_2_VO_12_ can be mainly attributed to librational and translational vibrations of the elements in A-O-M linkages accompanied by distortion of the polyhedra. First principles calculation for the phonon density of states and corresponding Grüneisen parameters in Y_2_Mo_3_O_12_ showed that 75 out of 111 low wavenumber phonons have negative Grüneisen parameters and contribute to the NTE. The lowest frequency optical branch (34.5 cm^−1^) having the largest negative Grüneisen parameter arises from the anharmonic translational vibrations of the Y and M in the plane of the nonlinear Y–O–Mo linkage, making the linkage become more bended and the Y and Mo atoms become closer. Dynamic simulation on connected YO_6_-O-MoO_4_ polyhedra revealed that the YO_6_ octahedron and MoO_4_ tetrahedron distorted unevenly as the temperature increases owing to the differences in vibrational directions and amplitudes of the oxygen atoms relative to Y or Mo atom, leading to both polyhedra to be closer and folded up. The NTE in ZrScMo_2_VO_12_ can be understood by the distortional quasi-rigid unit modes (QRUM) with nonzero vibrational frequencies as the polyhedra have similar connection as in Y_2_Mo_3_O_12_^39^,^40^.

**Figure 5:**
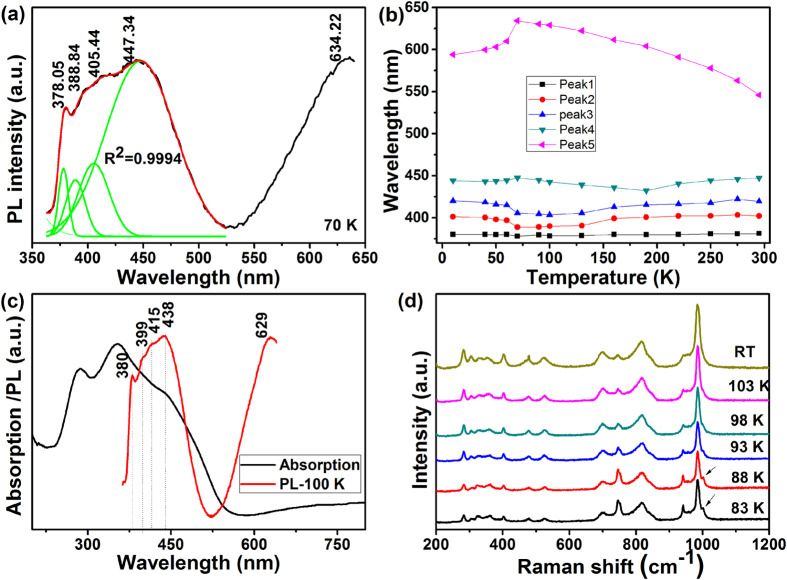
PL spectrum deconvolution, UV-vis absorption spectrum at RT and low temperature Raman spectrum. (**a**) PL spectrum deconvolution into three narrow and two broad bands; (**b**) Shift of the PL peak positions with temperature; (**c**) UV-vis absorption spectrum at RT and PL spectrum at 100 K; (**d**) Low temperature Raman spectra of ZrScMo_2_VO_12_.

For the optical property, the temperature-dependent PL spectra show several features: (1) The PL intensity increases with increasing temperature from 90 to 295 K, which is in contrast to that in a material with positive thermal expansion, while it decreases with increasing temperature from 10 to 70 K as usually observed due to increased phonon emissions and thus reduced transition probability; (2) The PL spectra can be deconvoluted into three narrow and two broad bands (Fig. 5a). The three narrow bands are close to the band edge and shift a little with temperature. Nevertheless, the two broad PL bands which are located at longer wavelengths shift obviously and oppositely with decreasing temperature. The one at the lowest energy shifts obviously to red until 70 K (545 nm at RT to 635 nm at 70 K) and then to blue below 70 K (Fig. 5b). Figure 5c shows the absorption spectrum of ZrScMo_2_VO_12_ at RT and the PL at 100 K. It is revealed that there is an Urbach tail near the band edge, which originates from localized excitons.

The abundance of physical properties in ZrScMo_2_VO_12_ could be understood by the particular design of the structure. ZrScMo_2_VO_12_ can be regarded as a structure modification of Sc_2_Mo_3_O_12_ by replacing one Sc^3+^ with Zr^4+^ and simultaneously replacing one Mo^6+^ with a V^5+^ in order to keep the balance of charge. Such substitutions have the function of n- and p-type like co-doping in semiconductors. Because a unit cell consists of four molecular units, and there are four pairs of the n- and p-type ions within one unit cell. The n- and p-type like co-doped ions occupy the center positions of the octahedra and tetrahedra in ZrScMo_2_VO_12_ and hence are highly localized. The n-type and p-type like doped ions creates donor and acceptor states in the band gap. Besides, they may also bind excitons, resulting in bound exciton complexes such as D^o^X, A^o^X, etc. The three narrow PL bands near the band edge are tentatively assigned to the inter-band transitions and the transitions from a donor-like state to the valence band (VB), and from the conduction band (CB) to an accepter-like state, respectively. The two broad PL bands which are dependent of temperature can be mainly attributed to the transitions from the energy levels of donor-acceptor pairs (DAP) to the VB.

The interaction between the donor and acceptor in the pairs is dependent of their separation. There exist DAPs with different separations due to that they are localized in different centers of octahedra and tetrahedra in the lattice. The DAPs with different separations have different interaction strengths, which should account for the broadness of the two broad PL bands.

The shift behavior of DAP peaks with temperature could be understood by the following Eq. (1) 41,


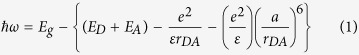


where *E*_*D*_ and *E*_*A*_ are the donor and acceptor levels measured from bottom of the CB and from the top of VB, respectively, *ε* is dielectric constant, *a is* the effective Van der Waals coefficient for the interaction between neutral donor and neutral acceptor and *r*_*DA*_ is the distance between neutral donor and acceptor, respectively. A blue shift of the corresponding PL peak occurs when *r*_*DA*_ decreases with reducing temperature. Otherwise a red shift happens. The opposite behaviors of the two broad PL bands are attributed to anisotropic thermal expansion property of the material. The separations of the DAPs oriented along the *b-* and *c*-axes become larger with decreasing temperature, leading to red shift of the PL peak, while those along the *a*-axis becomes shorter, resulting in a blue shift of the corresponding PL.

The shift trends of the PL peaks show a turning point around about 70 K. This is attributed to a phase transition of ZrScMo_2_VO_12_. In order to confirm this assumption, we measured the temperature dependent Raman spectra of ZrScMo_2_VO_12_ as shown in Fig. 5d. It is found that a Raman band at about 1002 cm^−1^ emerges gradually around 93 K and becomes obvious around 88 K, which indicates an orthorhombic to monoclinic phase transition^8^,^9^. The Raman spectra reveal that the phase transition is a sluggish process which starts at about 93 K and possibly not completed until 80 K or even lower as revealed by the successive increase of the Raman mode at about 1002 cm^−1^. Unfortunately, we are unable to get a Raman spectrum below 83 K with present accessories. The material should already be in a monoclinic phase at 70 K. Distinct changes of the PL take place only after complete phase transition while Raman spectra is sensitive to the phase transition process.

Materials of A_2_Mo_3_O_12_ family may crystallize either in monoclinic or orthorhombic structure depending on the A^3+^ cation size. Those with larger A^3+^ (A = Lu, Er, Yb and Y) cation size crystallize in an orthorhombic structure and are highly hygroscopic and NTE can only be observed after complete removal of crystal water^9^,^14^. The same happens for ZrMgW_3_O_12_^20^. On the other hand, those with smaller A^3+^ cation size crystallize in a monoclinic structure and transform to orthorhombic structure at elevated temperatures (above 473, 780, 658, 610 K for A = Al, Fe, Cr and In, respectively)^11^,^13^ and the NTE is only possible at the high temperature phase. The same happens for ZrV_2_O_7_ (above 473 K)^22^ and HfMgW_3_O_12_ (above 400 K)^19^. It is evident that ZrScMo_2_VO_12_ is a material with NTE at least from 150 to 823 K, covering RT and without hygroscopicity. To our knowledge, there were few reports on the PL property of NTE materials. Macalik *et al*^42^. reported the PL from Eu^3+^-doped Al_2_(WO_4_)_3_ while Naruke and Obaid^43^ reported up-converted emission in Tm^3+^ and Yb^3+^ doped monoclinic Gd_2_W_3_O_12_ and orthorhombic Lu_2_W_3_O_12_ with 980 nm laser excitation. In both reports, only the PLs from Eu^3+^ ions or up-converted emissions from Tm^3+^ and Yb^3+^ were observed while no obvious emissions from the host NTE materials were seen. Both of the hydrate and anhydrate orthorhombic phases are intrinsically low luminescent even if the Tm^3+^ and Yb^3+^ concentrations are proper^43^. This is due probably to the indirect band gap nature of the NTE material in which direct transition is not allowed. The intense PL in ZrScMo_2_VO_12_ is an intrinsic nature of the material itself and suggests a direct band gap property.

In summary, we developed a novel ZrScMo_2_VO_12_ material, which possesses excellent NTE property over a wide temperature range and exhibits intense wide-band photoluminescence covering the entire visible light region. Structure analysis shows that ZrScMo_2_VO_12_ crystallizes an orthorhombic structure with group of *Pbcn* (No. 60) at RT. A phase transition of this material between 70 and 90 K is revealed. The NTE property is attributed to the particular flexible and easily twisted polyhedra in the building blocks or the lateral anharmonic vibrations of the bridging oxygen. The intense white light emission possibly originates from the n- and p-type like co-doping effect which creates not only the donor- and acceptor-like states in the band gap, but also donor-acceptor pairs and even bound exciton complexes. Its excellent NTE property integrated with the intense white light emission functionality suggests potential applications of this material in LED and other photoelectric devices. The design strategy of this material could open a new avenue for developing functional NTE materials.

## Methods

### Sample Preparation

Analytical grade chemicals of ZrO_2_, Sc_2_O_3_, MoO_3_ and V_2_O_5_ were used as starting materials and mixed according to the molar ratio of Zr:Sc:Mo:V = 1:1:2:1. The raw materials were thoroughly mixed and ground for 2 h in an agate mortar. The homogenized raw materials were pressed into cylindrical pellets with diameter of 10 mm and thickness of 5 mm. The pellets within a lid-covered crucible were put into a tubular furnace preheated to sintering temperature at 973 K and maintained for 5 h and cooled down slowly to room temperature.

### Relative length change measurement

The relative length changes were measured with dilatometers (LINSEIS DIL L76 for high temperature and LINSEIS DIL L75 for low temperature).

### Temperature dependent X-ray diffraction measurements

High temperature X-ray powder data were collected on a Bruker D8 Advance X-ray diffractometer with a sealed tube X-ray generator (40 kV, 40 mA), Cu Kα (λ = 0.15405 nm) as radiation source. An mri MTC-furnace A2FA5 high-temperature attachment was used to control temperature and an AlCr heater was used as the heating stage. The sample was heated at a rate of 10 K/min and remained at each measurement temperature for 5 min before measurement. The crystal cell structure and crystal constants were obtained by the Rietveld refinement of XRD patterns with software of TOPAS 4.0.

### Differential scanning calorimetry and thermogravimetric measurements

Differential scanning calorimetry and thermogravimetric analysis were done on a Netzsch STA (Model 449F3) in the temperature range of 300–873 K with heating and cooling rates of 10 K/min.

### Temperature dependent Raman measurements

Temperature dependent Raman spectra were recorded by a Renishaw inVia Raman spectrometer with 532 nm excitation in the temperature range of 83–293 K. The temperature was controlled by a Linkam THMS600 Heating and Freezing Stage (Japan Hightech), and the cooling rate was 5 K/min.

### Photoluminescence measurements

The PL spectra from 295 K to 10 K were measured by a Fluoromax-4 spectrofluorometer (HORIBA Jobin Yvon), with a LakeShore 325 temperature controller.

## Additional Information

**How to cite this article**: Ge, X. *et al.* Negative thermal expansion and broad band photoluminescence in a novel material of ZrScMo_2_VO_12_. *Sci. Rep.* **6**, 24832; doi: 10.1038/srep24832 (2016).
